# Accurate infarct-size measurements from accelerated, compressed sensing reconstructed cine-MRI images in mouse hearts

**DOI:** 10.1186/1532-429X-14-S1-P57

**Published:** 2012-02-01

**Authors:** Tobias Wech, Debra Medway, Craig A Lygate, Stefan Neubauer, Herbert Köstler, Jurgen E Schneider

**Affiliations:** 1 Institute of Radiology, University of Würzburg, Würzburg, Germany; 2 Cardiovascular Medicine, University of Oxford, Oxford, UK

## Summary

Three-fold accelerated cine-MRI followed by Compressed Sensing reconstruction has been shown to provide accurate measurement of cardiac functional parameters in normal and chronically infarcted mouse hearts. This study demonstrates that infarct size can also be accurately quantified from the three-fold undersampled cine-data.

## Background

Compressed Sensing (CS) is becoming more common as an alternative means to speed up the inherently time-consuming data acquisition process in Magnetic Resonance Imaging (MRI). More specifically, global cardiac function (cine) MRI combined with CS has been demonstrated to provide a three-fold reduction in scan time in normal mice and in a murine model of chronic myocardial infarction without compromising the accuracy of cardiac functional parameters (Wech et al, JMRI 2011). However, this study did not validate the measurement of infarct size, an important parameter that is also obtained from these data and does not require the administration of exogenous contrast agents. We therefore sought to investigate whether or not three-fold undersampled and CS-reconstructed cine-images allow for an accurate determination of infarct size.

## Methods

Mice with permanent ligation of the left coronary artery were scanned on a preclinical 9.4 T MR system with a standard cine-MRI protocol 8 weeks post surgery. The fully acquired cine-data sets were retrospectively undersampled by a factor of three and subjected to the CS reconstruction described in Wech et al (JMRI 2011). Using the method proposed by Takagawa et al (J Appl Physiol 2007), infarct size was subsequently measured in fully and undersampled data by an operator, who was blinded to animal ID and acquisition/reconstruction schemes.

## Results

**Figure 1:**
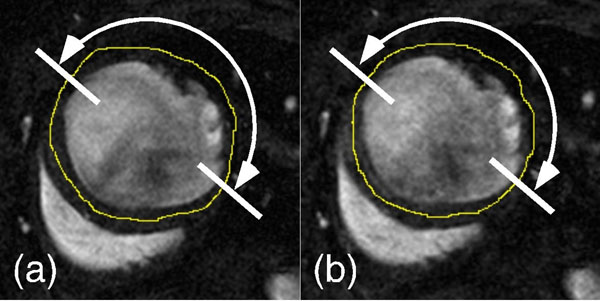


Figure 1 depicts (a) fully and (b) three-fold undersampled end-diastolic frames of a mid-ventricular slice across the same mouse heart. The contours for the midline (yellow line) and the akinetic part of the myocardium (white arrows) are shown. Excellent agreement in infarct size was found between the two groups (40.3 ± 6.1% vs. 40.3 ± 6.5% - mean ± SD, fully vs. three-fold undersampled, p = 0.91, n = 3), suggesting that the image quality is sufficient to accurately derive infarct size.

## Conclusions

Our retrospective analysis demonstrates that an undersampling factor of three does indeed allow for an accurate determination of infarct size. Work is in progress to validate this approach further using a wider range of infarct sizes.

## Funding

This work was funded by the British Heart Foundation and the Elite Network of Bavaria.

